# miR-203 Inhibits Cell Proliferation and Migration of Lung Cancer Cells by Targeting PKCα

**DOI:** 10.1371/journal.pone.0073985

**Published:** 2013-09-10

**Authors:** Chen Wang, Xueliang Wang, Hongwei Liang, Tao Wang, Xin Yan, Minghui Cao, Nan Wang, Suyang Zhang, Ke Zen, Chenyu Zhang, Xi Chen

**Affiliations:** 1 Jiangsu Engineering Research Center for microRNA Biology and Biotechnology, State Key Laboratory of Pharmaceutical Biotechnology, School of Life Sciences, Nanjing University, Nanjing, Jiangsu, China; 2 Department of Cardio-Thoracic Surgery, Nanjing Drum Tower Hospital, the Affiliated Hospital of Nanjing University Medical School, Nanjing, Jiangsu, China; 3 Tianjin Medical University Cancer Institute and Hospital, Tiyuanbei, Tianjin, China; Sun Yat-sen University Medical School, China

## Abstract

PKCα (protein kinase C alpha, PRKCA) is an important protein involved in several steps of signaling pathways in lung cancer, and microRNAs (miRNAs) have also been shown to participate in lung carcinogenesis. However, it is not clear how PKCα and miRNAs are correlated in the disease. In this report, we aimed to identify novel miRNAs that target PKCα and to study their biological function. Using bioinformatics analysis, we predicted one novel candidate, miR-203, and found differential expression patterns of miR-203 and PKCα in human lung cancer tissues. Moreover, we experimentally validated miR-203 as a direct regulator of PKCα. Finally, we demonstrated that the targeting of PKCα by miR-203 played a critical role in regulating cell proliferation, apoptosis and migration in lung cancer cells. In summary, this study identifies a novel miRNA that targets PKCα and illustrates that the downregulation of PKCα by miR-203 modulates biological processes in lung cancer cells.

## Introduction

Lung cancer is the leading cause of cancer-related deaths worldwide, and non–small cell lung cancer (NSCLC) accounts for approximately 80% of all cases [1]. The majority of lung cancers (56%) are diagnosed at a distant stage because early disease is typically asymptomatic; only 15% of cases are diagnosed at a local stage [2]. Indeed, patients with lung cancer often exhibit tumor cell invasion and metastasis before diagnosis which renders current treatments, including surgery, radiotherapy, and chemotherapy ineffective. The overall 5-year survival rate for non-small cell lung cancer is very low (17.1%). Therefore, studying the molecular basis of lung cancer is crucial for designing new therapeutic agents that will improve the survival rate.

Protein kinase C (PKC) is a serine/threonine kinase that plays a key role in several signal transduction pathways, including those involved in cellular proliferation, differentiation, and apoptosis [3–5]. The PKC family contains 10 related isoforms with different cofactor requirements, tissue and subcellular distribution, and substrate specificity [6]. The family is divided into conventional (cPKCs: α, βI, βII, and γ), novel (nPKCs: δ, ε, η, and θ), and atypical (aPKCs: ζ and ι/λ) subclasses. PKC, including PKCα (PRKCA), plays a part in lung cancer. The level of PKCα protein is significantly higher in NSCLC cell lines (H1355, A549, H1703, H157, and H1155) when compared to primary human lung epithelial cells (NHBE); therefore, increased PKCα expression may be a general feature of NSCLC cells [7]. There have been several reports on the role of PKCα in cellular proliferation, apoptosis and the migration of lung cancer cells. PKCα has been shown to bind and phosphorylate the scaffold protein discs large homolog 1 (DLG1) and promote cell migration in NSCLC cells [8]. Additionally, the suppression of PKCα enhances the cytotoxicity and mutagenicity of lead acetate (Pb)-treated CL3 human lung cancer cells [9]. Staurosporine, a potent PKC inhibitor, controls cell adhesion, mobility, and invasion of A549 cells [10]; IL1-beta induces the expression of urokinase plasminogen activator (uPA) via PKCα, which leads to the migration of A549 NSCLC cells [11].

microRNAs (miRNAs) are critical regulators of gene expression [12,13]. Mature miRNAs bind target mRNAs at complementary sites in the 3′-untranslated regions (3′-UTRs) or in the coding sequences, thereby suppressing the expression of the target gene [14,15]. miRNAs are deregulated in human lung cancer and play important roles in carcinogenesis [16]. For example, low expression of let-7a and high expression of the miR-17-92 cluster are associated with a poor clinical outcome in lung cancer [17,18]. The miR-34 family is also repressed in cancer and is involved in p53-associated tumor suppression in many cancers [19–23], including lung cancer [24]. These findings underscore the need for an in-depth search for miRNAs aberrantly expressed during lung carcinogenesis that may play critical roles in regulating lung cancer growth or tumorigenesis.

Although the deregulation of miRNAs and PKCα play important roles in lung carcinogenesis, no correlation between PKCα and miRNAs has been reported. In this study, we look for miRNAs that could target PKCα and influence cellular function.

## Materials and Methods

### Ethics statement

The lung cancer and matched normal adjacent tissue samples were derived from patients undergoing a surgical procedure at Nanjing Drum Tower Hospital (Nanjing, China). All of the patients or their guardians provided written consent and the Ethics Committee from the Nanjing University and Nanjing, Drum Tower Hospital approved all aspects of this study. Tissue fragments were immediately frozen in liquid nitrogen at the time of surgery and stored at -80 °C. Frozen tissues were homogenized and total RNA was extracted using TRIzol Reagent (Invitrogen, Carlsbad, CA, USA) according to the manufacturer’s instructions. The clinical features of the patients are listed in Table 1.

**Table 1:** The clinical features of lung cancer patients.

	Age	Gender	Pathological Stage	Tumor Hystotype
1	48	M	IIIA (T2b, N2, cMo)	Squamous Cell Carcinoma
2	70	F	IA (T1b, No, cMo)	Adenocarcinoma
3	62	F	IIB (T3, No, cMo)	Adenocarcinoma
4	55	F	IIIA (T3, N2, Mo)	Adenocarcinoma
5	67	M	IB (T1, No, Mo)	Adenocarcinoma
6	49	M	IV (T4, N2, M1a)	Adenocarcinoma

### Cells, reagents, and antibodies

Human lung adenocarcinoma A549 cells were purchased from the China Cell Culture Center (Shanghai, China). The cells were maintained in Dulbecco’s Modified Eagle Medium (DMEM; Gibco, CA, USA) supplemented with 10% fetal bovine serum (FBS; Gibco) and were grown at 37 °C in a humidified atmosphere with 5% CO_2_. Synthetic RNA molecules, including the pre-miR-203, and the scrambled non-coding RNA (ncRNA) were purchased from Ambion (Austin, TX, USA). Anti-PKCα (H-7) and anti-GAPDH (6C5) antibodies were obtained from Santa Cruz Biotechnology (CA, USA). Cell Counting Kit-8 was purchased from Dojindo (Japan). The FITC-Annexin V Apoptosis Detection Kit I was obtained from BD Biosciences (CA, USA).

### miRNA and siRNA transfection

miR-203 overexpression was achieved by transfecting cells with pre-miR-203, a synthetic RNA oligonucleotide that mimics the miR-203 precursor, and a ncRNA served as a negative control. Three siRNA sequences targeting different sites of human PKCα cDNA (si-PKCα) were designed and synthesized by Invitrogen. A scrambled siRNA that did not target human PKCα cDNA was included as a negative control. siRNA sequences were as follows: si-PKCα #1: 5’-GGAUGUGGUGAUUCAGGAU-3’ (sense); si-PKCα #2: 5’-GCAAAGGACUGAUGACCAA-3’ (sense); si-PKCα #3: 5’-AAGCUCCAUGUCACAGUACGA-3’ (sense).

A549 cells were seeded in 6-well plates and were transfected the following day using Lipofectamine 2000 (Invitrogen), according to the manufacturer’s instructions. For each well, equal doses (100 pmol) of scrambled ncRNA, pre-miR-203, scrambled siRNA, or si-PKCα were added. Cells were harvested 24 h after transfection. The expression level of miR-203 was analyzed by quantitative RT-PCR, while PKCα protein level was assessed by Western blot using an antibody against PKCα. These samples were normalized by blotting with an antibody against GAPDH. The ImageJ software was used to quantify the protein levels. The siRNA sequence with the best interfering effect (si-PKCα #3) was selected and used in further studies.

### Overexpression of PKCα

A mammalian expression plasmid encoding the human PKCα open reading frame without 3’-UTR was purchased from Invitrogen. An empty plasmid served as a negative control. The PKCα overexpression plasmid was transfected into A549 cells using Lipofectamine 2000 (Invitrogen) according to the manufacturer’s instructions.

### RNA isolation and quantitative RT-PCR

Total RNA was extracted from the cultured cells using TRIzol Reagent (Invitrogen) according to the manufacturer’s instructions. For quantitative RT-PCR analysis of PKCα and β-actin transcripts, 1 μg total RNA was reverse transcribed into cDNA using an oligdT and Thermoscript Reverse Transcriptase (TaKaRa, Dalian, China). Real-time PCR for the PKCα and β-actin transcripts was performed on an Applied Biosystems 7300 Sequence Detection System (Applied Biosystems, Foster City, CA, USA) using SYBR green dye (Invitrogen). PCR reactions were performed in a 20 μL reaction including 1μL cDNA, 1× QuantiTect SYBR green PCR Master Mix, and 0.5 μM sense and antisense primers. The reactions were incubated in a 96-well plate at 95 °C for 5 min, followed by 40 cycles of 95 °C for 30 s, 60 °C for 30 s, and 72 °C for 30 s. All reactions were run in triplicate. After the reactions were run, the threshold cycles (C_T_) were determined using fixed threshold settings. The sequences of the sense and antisense primers used for the amplification of PKCα and β-actin were as follows: PKCα (sense): 5’-GTGGCAAAGGAGCAGAGAAC-3’; PKCα (antisense): 5’-TGTAAGATGGGGTGCACAAA-3’; β-actin (sense): 5'-AGTACTTCCTC TGCCCTGCTGCAG-3'; β-actin (antisense): 5'-AGGGCAGGCAGCGTATATACAGGA-3'.

Assays to quantify mature miR-203 were carried out using Taqman microRNA probes (Applied Biosystems) according to the manufacturer’s instructions. Briefly, 1 μg total RNA was reverse-transcribed into cDNA using AMV Reverse Transcriptase (TaKaRa) and a stem-loop RT primer (Applied Biosystems). Real-time PCR was performed using a TaqMan PCR kit on an Applied Biosystems 7300 Sequence Detection System (Applied Biosystems). All reactions, including the no template controls, were run in triplicate. After the reactions, the C_T_ values were determined using fixed threshold settings. In the experiments presented here, miRNA expression in the cells was normalized to the expression of the U6 snRNA. The relative amount of miR-203 to the internal U6 control was calculated using the equation 2^-∆CT^, where ΔC_T_ = C_T miR-203_ -C_T U6_.

### miRNA target prediction

The miRNAs that may target PKCα were determined using algorithms from TargetScan (http://genes.mit.edu/targetscan/) [25], PicTar (http://pictar.bio.nyu.edu/) [26], and miRanda (http://cbio.mskcc.org/cgi-bin/mirnaviewer/mirnaviewer.pl) [27].

### Plasmid construction and luciferase assay

A partial sequence of the human PKCα 3’-UTR, which includes the predicted miR-203 binding sites, was synthesized by Invitrogen, with an additional 3'-phosphorylation modification. The sequence was as follows: PKCα 3’-UTR (sense): 5’-CTAGTTCTAAGGACGTTGCTGAACAAGCGTGTGAAATCATTTCAGATCAAGGATAAGCCAGTGTGTACATATGTA-3’; PKCα 3’-UTR (antisense): 5’-AGCTTACATATGTACACACTGGCTTATCCTTGATCTGAAATGATTTCACACGCTTGTTCAGCAACGTCCTTAGAA-3’. A double stranded molecule was formed by annealing these two single chains at 60°C, and this duplex was inserted into the p-MIR-report plasmid (Ambion). Efficient insertion was confirmed by sequencing. For the luciferase reporter assays, cells were cultured in 6-well plates, and each well was transfected with 2 μg firefly luciferase reporter plasmid, 2 μg β-galactosidase expression vector (Ambion), and equal amounts of scrambled ncRNA or pre-miR-203 using Lipofectamine 2000 (Invitrogen). The β-galactosidase vector was used as a transfection control. Cells were assayed using the luciferase assay kits (Promega, Madison, WI, USA) 24 h after transfection. The reported data represent three independent experiments.

### Cell viability assay

The viability of A549 cells was determined using the Cell Counting Kit-8 (Dojindo), according to the manufacturer’s instructions. Briefly, A549 cells were plated at 5.0×10^3^ cells per well in 96-well plates and incubated overnight in DMEM medium supplemented with 10% FBS. After transfection, 10 µl CCK-8 liquid was added to the test well and incubated for 3 h. Absorbance (*A*) was then measured at a wavelength of 450 nm.

### Cell migration assay

The migration ability of A549 cells was tested using a Transwell Boyden Chamber (6.5 mm, Costar, Cambridge, MA). Cells were treated with ncRNA, pre-miR-203, or siRNAs for 6 h and were suspended in serum-free DMEM medium at a concentration of 4×10^5^ cells/mL; then, 4×10^4^ cells/well was added to the upper chamber. Simultaneously, 0.5 mL DMEM supplemented with 10% FBS was added to the lower compartment, and the transwell-containing plates were incubated for 18 h in a 5% CO_2_ atmosphere saturated with H_2_O. At the end of the incubation, the cells that had entered the lower surface of the filter membrane (migrant cells) were fixed with 4% paraformaldehyde for 15 min at room temperature; the cells were then washed three times with distilled water and stained with 0.1% crystal violet for 15 min at room temperature. The cells remaining on the upper surface of the filter membrane (non-migrant) were gently scraped off with a cotton swab. Images of migrant cells were captured using a photomicroscope (BX51, Olympus, Japan). Cell migration was quantified by blind counting of the migrated cells on the lower surface of the membrane; five fields were counted per chamber.

### Cell apoptosis assay

Twenty-four hours after transfection with ncRNA, pre-miR-203, or siRNA, A549 cells were treated with 200 μM hydrogen peroxide (H_2_O_2_) for 30 min to induce apoptosis. As per the manufacturer’s instructions of the FITC-Annexin V Apoptosis Detection Kit I (BD Biosciences), the cells were then washed twice with cold PBS and resuspended in 1× binding buffer at a concentration of 1×10^6^ cells/mL. Cells (1×10^5^ cells) were transferred to a 5 mL culture tube, and FITC-Annexin V and propidium iodide (PI) were added. The cells were incubated for 15 min at room temperature in the dark and were analyzed by flow cytometry (BD Biosciences) within 1 h of staining.

### Statistical analysis

All photos of Western blots, cell apoptosis assays, and migration assays were representative of at least three independent experiments. Data shown are presented as the mean ± standard deviation (S.D.). A 2-tailed Student’s *t* test was used for comparisons, and a *p* value of <0.05 was considered significant.

## Results

### Prediction of miRNAs that can target PKCα

With the help of three miRNA target prediction programs (TargetScan, PicTar and miRanda), we predicted that miR-203 targets the PKCα mRNA transcript.

**Figure 1:**
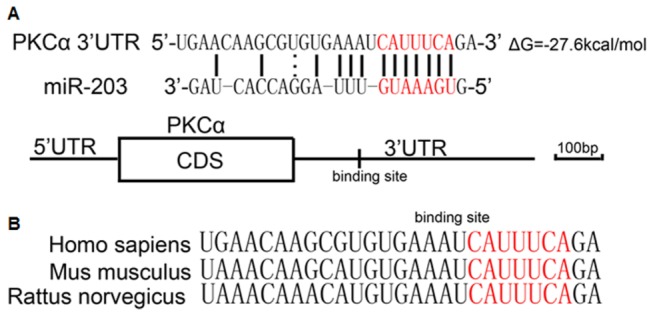
Identification of the conserved miR-203 binding sites within the PKCα mRNA 3’-UTR. A, schematic description of the hypothesized duplexes formed by the interactions between the PKCα 3’-UTR binding sites and miR-203. The predicted structure of the base-paired hybrid is diagrammed. Paired bases are indicated by a *black line*, and G:U pairs are indicated by *three dots*. The predicted free energy of the hybrid is indicated. B, sequence alignment of the putative miR-203 binding sites across species. The seed complementary sites are marked in *red*, and all nucleotides in the regions are conserved in several species, including human, mouse and rat.

The predicted interactions between miR-203 and its target sites in the PKCα 3’-UTR are illustrated in Figure 1A. As shown in this figure, there is one potential miR-203 target site in the 3’-UTR of the PKCα mRNA sequence. The minimum free energy values of these interactions are -27.6 kcal/mol, as determined by RNA hybrid analysis [28]. Moreover, perfect base pairing between the seed region (the core sequence that encompasses the first 2-8 bases of the mature miRNA) and the cognate targets was noted (Figure 1A), and the miR-203 binding sequences in the PKCα 3’-UTR are highly conserved across species (Figure 1B).

### Differential expression patterns of miR-203 and PKCα in human lung cancer tissues

**Figure 2:**
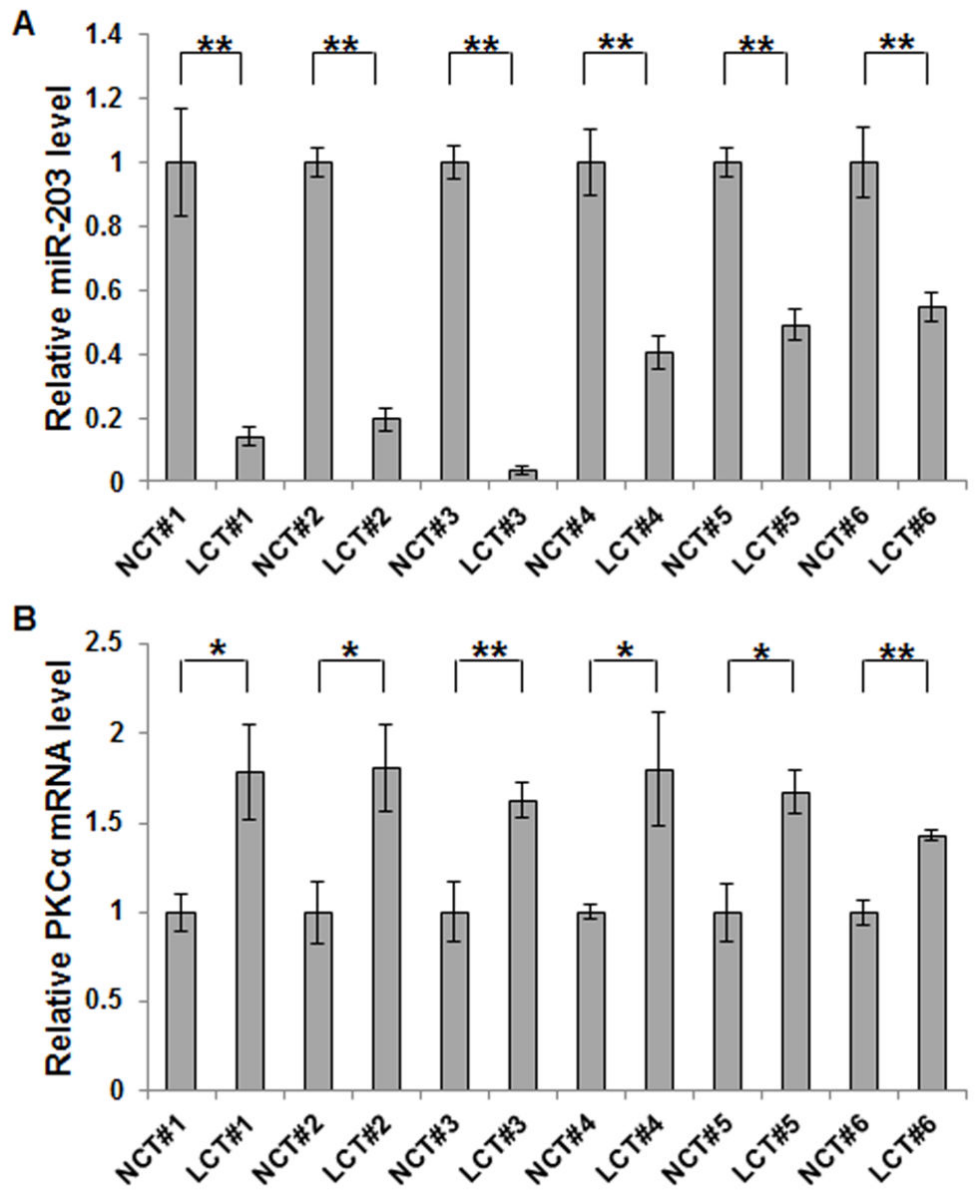
Differential expression patterns of miR-203 and PKCα in human lung cancer tissues. A, quantitative real-time PCR analysis of the relative miR-203 expression in 6 pairs of lung cancer tissue (LCT) and noncancerous tissue (NCT) samples. B, quantitative real-time PCR analysis of the relative PKCα mRNA level in the same 6 pairs of LCT and NCT samples. The results in A, and B are presented as the mean ± S.D. of three independent experiments (*, *p*<0.05; ** *p*<0.01).

miRNAs are generally thought to negatively regulate the expression of their targets through translational repression or mRNA degradation [29,30]. Therefore, if a miRNA mediates the degradation of its targeted mRNAs, this miRNA and its targets should have opposite expression patterns. Based on this, we examined the expression patterns of miR-203 and PKCα in the same 6 pairs of lung cancer and corresponding noncancerous tissue samples. Using quantitative real-time PCR analysis, we demonstrated that the expression of miR-203 was significantly lower in human lung cancer tissues than in the adjacent normal tissues (Figure 2A); in contrast, PKCα mRNA expression was noticeably higher in cancer cells (Figure 2B).

### Validation of PKCα regulation by miR-203

**Figure 3:**
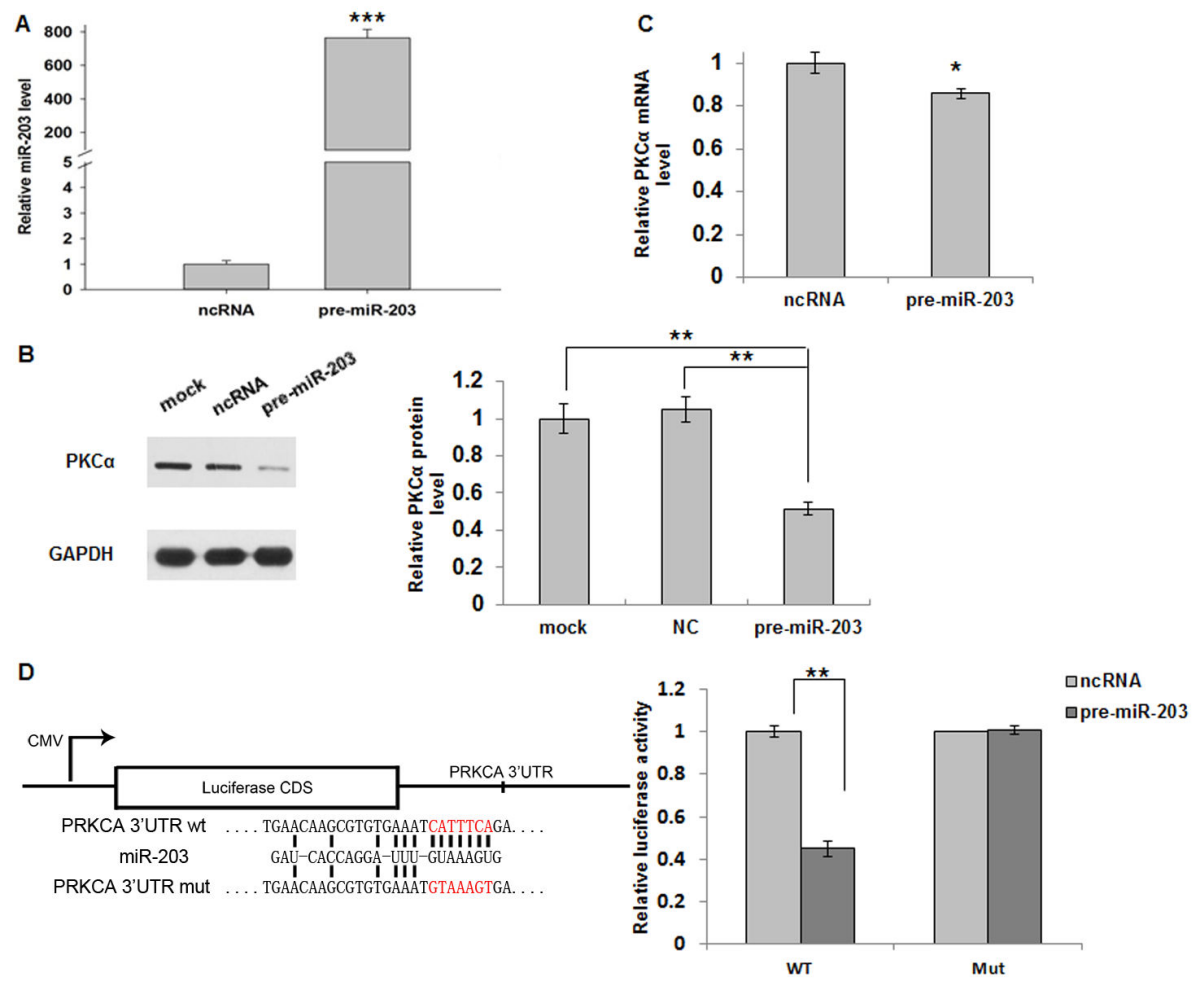
Regulation of PKCα expression by miR-203. A, overexpression of miR-203. A549 cells were seeded in a 6-well plate and transfected the following day. For each well, 100 pmol scrambled ncRNA or pre-miR-203 was added. The levels of miR-203 were evaluated by quantitative RT-PCR 24 h after transfection. For comparison, the expression levels of miR-203 in ncRNA-transfected cells were set at 1. The *y*-axis shows arbitrary units representing the relative miR-203 expression levels. The results are presented as the mean ± S.D. of three independent experiments (*** *p*<0.001). B, Immunoblot for endogenous PKCα protein in A549 cells that were either mock transfected or transfected with ncRNA or pre-miR-203 for 24 h. GAPDH was used as a loading control. Pictures of the Western blot assay were analyzed using the ImageJ software, and a statistical analysis is shown in the right panel (mean ± S.D.; ** *p*<0.01). C, quantitative RT-PCR analysis of PKCα mRNA levels in A549 cells treated with scrambled ncRNA or pre-miR-203. The *y*-axis shows relative PKCα mRNA levels normalized to the levels of β-actin (mean ± S.D.; *, *p*<0.05). D, direct recognition of the PKCα 3’-UTR by miR-203. Either wild-type (wt) or mutant (mut) miR-203 binding sites (the sequence that interacts with the 2-8 bases of miR-203 were mutated) in the PKCα 3’-UTR are depicted. Firefly luciferase reporters containing either the wild-type (wt) or the mutant (mut) human PKCα 3’-UTR were co-transfected into A549 cells along with scrambled ncRNA, or pre-miR-203. At 24 h post-transfection, the cells were assayed using a luciferase assay kit. Firefly luciferase values were normalized to β- galactosidase activity and plotted as relative luciferase activity. For comparison, the luciferase activity in the ncRNA-transfected cells was set as 1. The results are presented as the mean ± S.D. of three independent experiments (** *p*<0.01).

For further validation, human lung adenocarcinoma A549 cells were transfected with ncRNA or pre-miR-203, and the cells were analyzed for the expression of miR-203 by quantitative RT-PCR 24 h after transfection. All cells that were transfected with pre-miR-203 showed a significantly increased expression of the mature miR-203 (Figure 3A). To determine whether the overexpression of miR-203 had any effect on the levels of PKCα, we repeated the above experiments and examined the expression of PKCα protein and mRNA 24 h after transfection. As shown in Figure 3B, the expression of PKCα protein was significantly reduced by the introduction of pre-miR-203, whereas cells transfected with the scrambled ncRNA maintained a considerable amount of PKCα protein. Similarly, cells transfected with pre-miR-203 had decreased levels of PKCα mRNA, relative to cells transfected with the ncRNA (Figure 3C). In animals, miRNAs are believed to act mainly through translational repression rather than mRNA cleavage [30], but new studies show that metazoan miRNAs can reduce the levels of many of their target transcripts, not just the amount of protein deriving from these transcripts [31]. Our data suggest that miR-203 regulates the expression of PKCα at both the transcript and protein levels, and considering greater decrease in PKCα protein, it might act more on translational repression than mRNA degradation.

### PKCα is a direct target of miR-203

To determine whether the negative regulatory effects miR-203 exerted on PKCα expression were mediated through the binding of miR-203 to the presumed sites in the 3’-UTR of the PKCα mRNA, we fused part of the PKCα 3’-UTR, which includes the predicted miR-203 binding sites, downstream of the firefly luciferase reporter plasmid. The resulting plasmid was introduced into A549 cells along with a transfection control plasmid expression β-galactosidase and pre-miR-203 or the scrambled ncRNA. As expected, overexpression of miR-203 resulted in a significant decrease in the luciferase reporter activity, which was normalized to β- galactosidase activity, when compared to cells treated with the scrambled ncRNA (Figure 3D). Furthermore, we introduced point mutations into the corresponding seed complementary sites in the PKCα 3’-UTR to eliminate the predicted miR-203 binding site. As shown in Figure 3D, mutations in the complementary seed sites almost fully rescued the repression of the reporter activity caused by the expression of pre-miR-203. This suggests that the binding site strongly contributes to the miRNA: mRNA interaction that mediates the post-transcriptional inhibition of PKCα expression. In conclusion, our results demonstrate that miR-203 directly recognizes the 3’-UTR of the PKCα mRNA transcript and binds to it to downregulate its expression.

### The role of miR-203 mediated PKCα downregulation in cell proliferation, apoptosis, and cell migration

To investigate the cellular phenotypes triggered by the miR-203 mediated downregulation of PKCα, A549 cells were transfected with either pre-miR-203 or si-PKCα and analyzed for changes in cell proliferation, apoptosis and migration.

**Figure 4:**
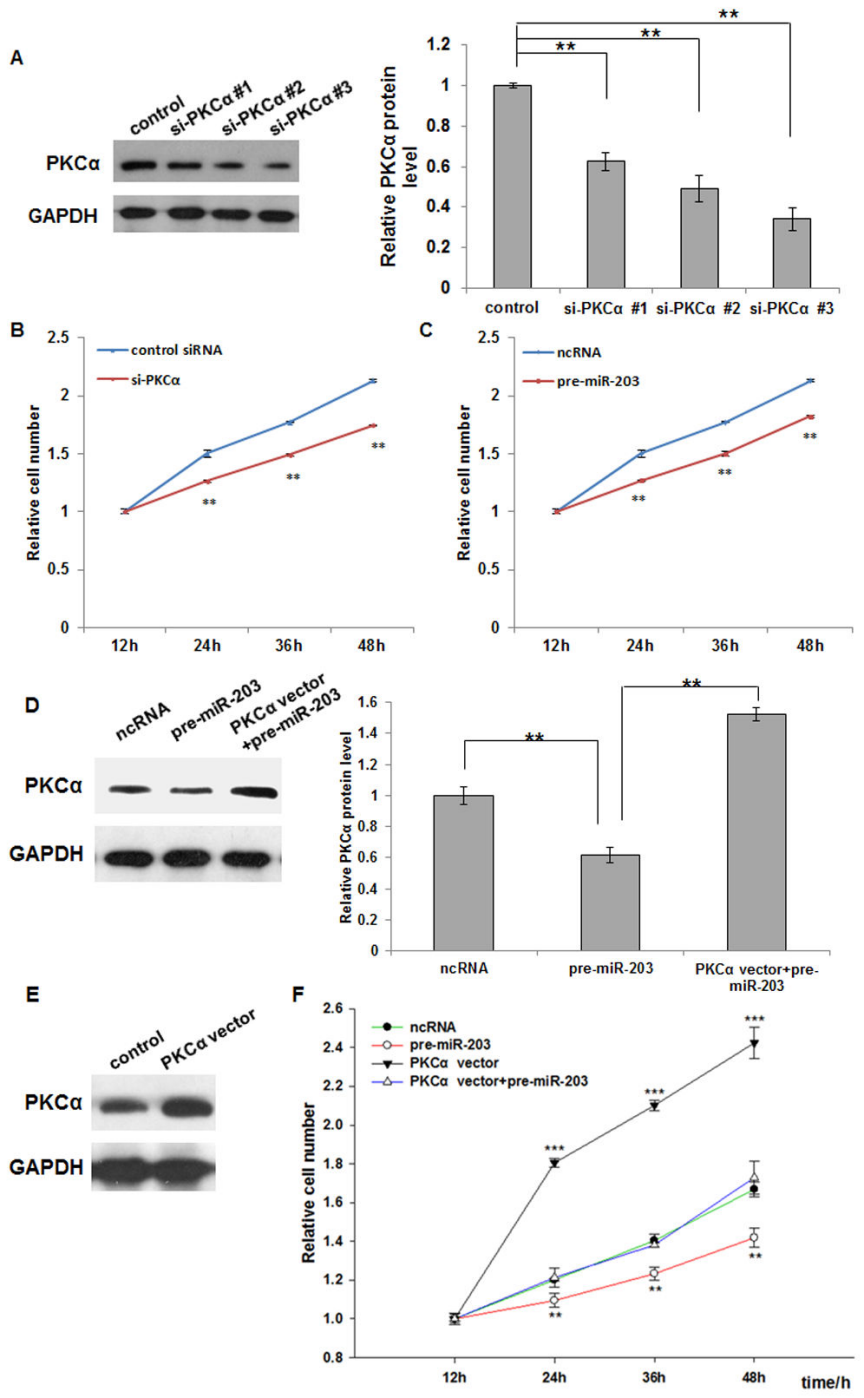
The role of the regulation of PKCα by miR-203 in cellular proliferation. A, validation of the siRNA against PKCα. Three siRNAs targeting different sites of human PKCα cDNA and a scrambled control siRNA were transfected into A549 cells using Lipofectamine 2000. Western blot analysis depicts PKCα protein levels at 24 h post-transfection. Normalized quantification of the immunoblots was carried out from independent experiments. Data are presented as the mean ± S.D. (** *p*<0.01). B, cell viability assay at 12, 24, 36, and 48 h after transfection of A549 cells with equal doses of control siRNA or si-PKCα, or C, equal doses of scrambled ncRNA or pre-miR-203 (mean ± S.D.; ** *p*<0.01). D, Western blot analysis of the protein level of PKCα in A549 cells transfected with ncRNA, pre-miR-203, or pre-miR-203 plus PKCα overexpression plasmid, and a statistical analysis is shown in the right panel (mean ± S.D.; ** *p*<0.01). E, Immunoblot for PKCα protein in A549 cells that were either mock transfected or transfected with PKCα overexpression plasmid. F, cell viability assay at 12, 24, 36, and 48 h after transfection of A549 cells with ncRNA, pre-miR-203, pre-miR-203 plus PKCα overexpression plasmid, or PKCα overexpression plasmid alone (mean ± S.D.; ** *p*<0.01; *** *p*<0.001).

As shown in Figure 4A, most efficient interference of PKCα expression could be achieved by si-PKCα #3 (named si-PKCα afterwards) transfection, compared to the control siRNA. We determined the proliferation rates of A549 cells with decreased expression of PKCα or overexpression of miR-203 using the Cell Counting Kit-8. In contrast with the control siRNA-transfected cells, cells transfected with si-PKCα proliferated at a significantly lower rate (Figure 4B). Furthermore, a significant difference was observed in the proliferation rates between the cells transfected with ncRNA and pre-miR-203 (Figure 4C).

Subsequently, we investigated whether overexpression of PKCα is sufficient to reverse the inhibitory effects of miR-203 on PKCα and biological processes in lung cancer cells. A plasmid designed to specially express the full-length open reading frame (ORF) of PKCα without the miR-203-responsive 3’-UTR was constructed and transfected into pre-miR-203 transfected A549 cells. Compared to cells transfected with pre-miR-203, the cells transfected with pre-miR-203 and the PKCα overexpression plasmid exhibited significantly higher levels of PKCα (Figure 4D), suggesting that miR-203-resistant PKCα rescued the PKCα suppression caused by miR-203. Cells transfected with the PKCα overexpression plasmid alone also showed more expression level of PKCα compared to cells transfected with an empty plasmid control (Figure 4E). Consequently, overexpression of PKCα rescued miR-203 mediated downregulation of the proliferation rates of A549 cells (Figure 4F). These results suggest that miR-203 might inhibit cell proliferation by silencing PKCα.

**Figure 5:**
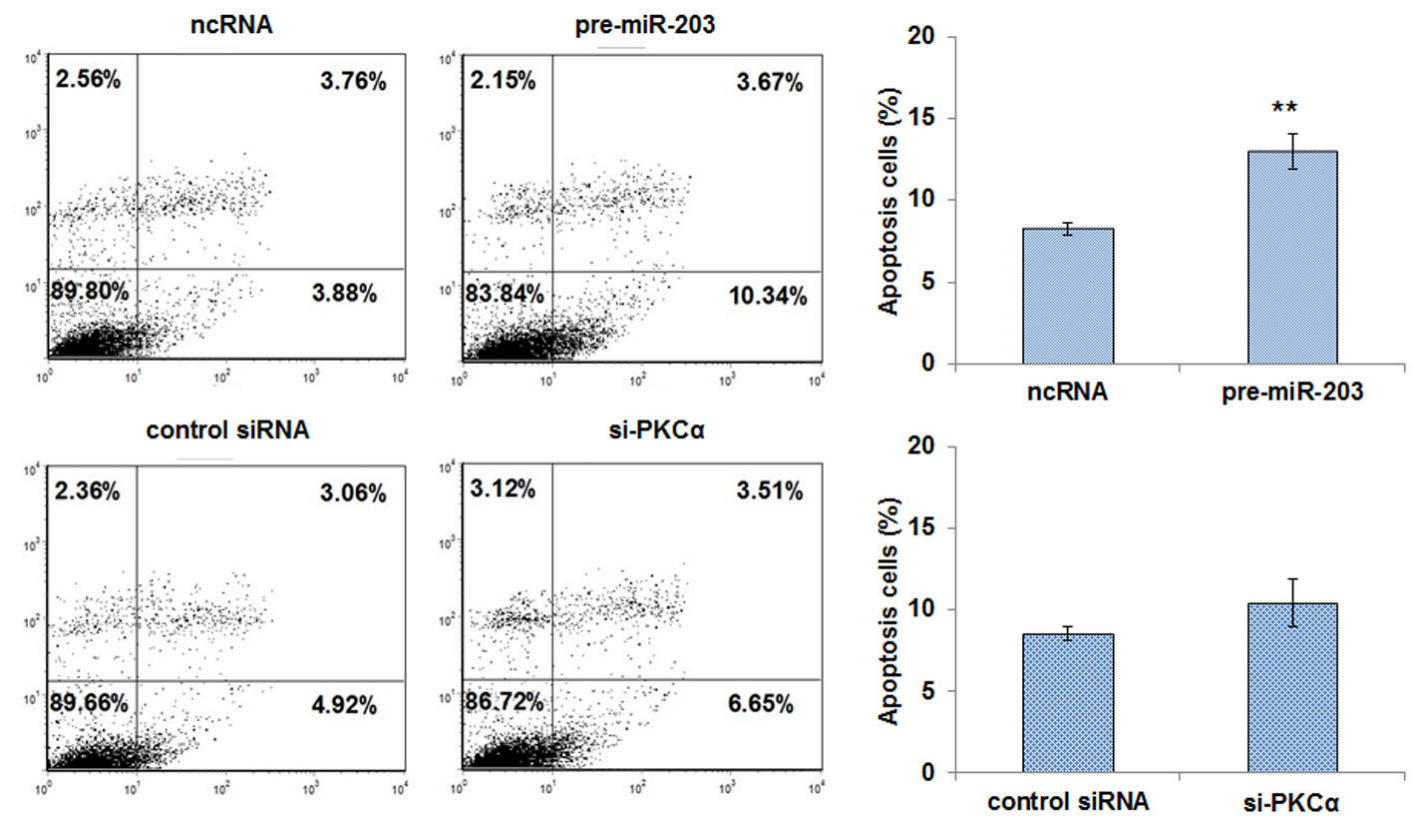
The role of PKCα regulation by miR-203 in apoptosis. A549 cells were transfected with equal doses of scrambled ncRNA or pre-miR-203, or equal doses of control siRNA or si-PKCα. Cell apoptosis profiles were analyzed by flow cytometry. The biparametric histogram shows cells in early (bottom right quadrant) and late apoptotic states (upper right quadrant). Viable cells are double negative (bottom left quadrant). The experiment was repeated three times, and a statistical analysis is shown in the right panel (mean ± S.D.; ** *p*<0.01).

After A549 cells were transfected with pre-miR-203, ncRNA, or si-PKCα for 24 h, they were treated with 200 μM hydrogen peroxide for 30 min to induce apoptosis. We then investigated apoptosis in cells with an increased miR-203 expression or silenced PKCα by flow cytometry analysis. When compared to cells transfected with ncRNA, the percentage of apoptotic cells in the pre-miR-203 transfection group was significantly higher, from 7.64% to 14.01%, respectively (Figure 5). When compared to cells transfected with control siRNA, transfection with si-PKCα slightly, but not significantly, increased the percentage of apoptotic cells, from 7.98% to 10.16%, respectively. These results suggest that miR-203 might promote cell apoptosis, but this effect only partially relies on its downregulation of PKCα.

**Figure 6:**
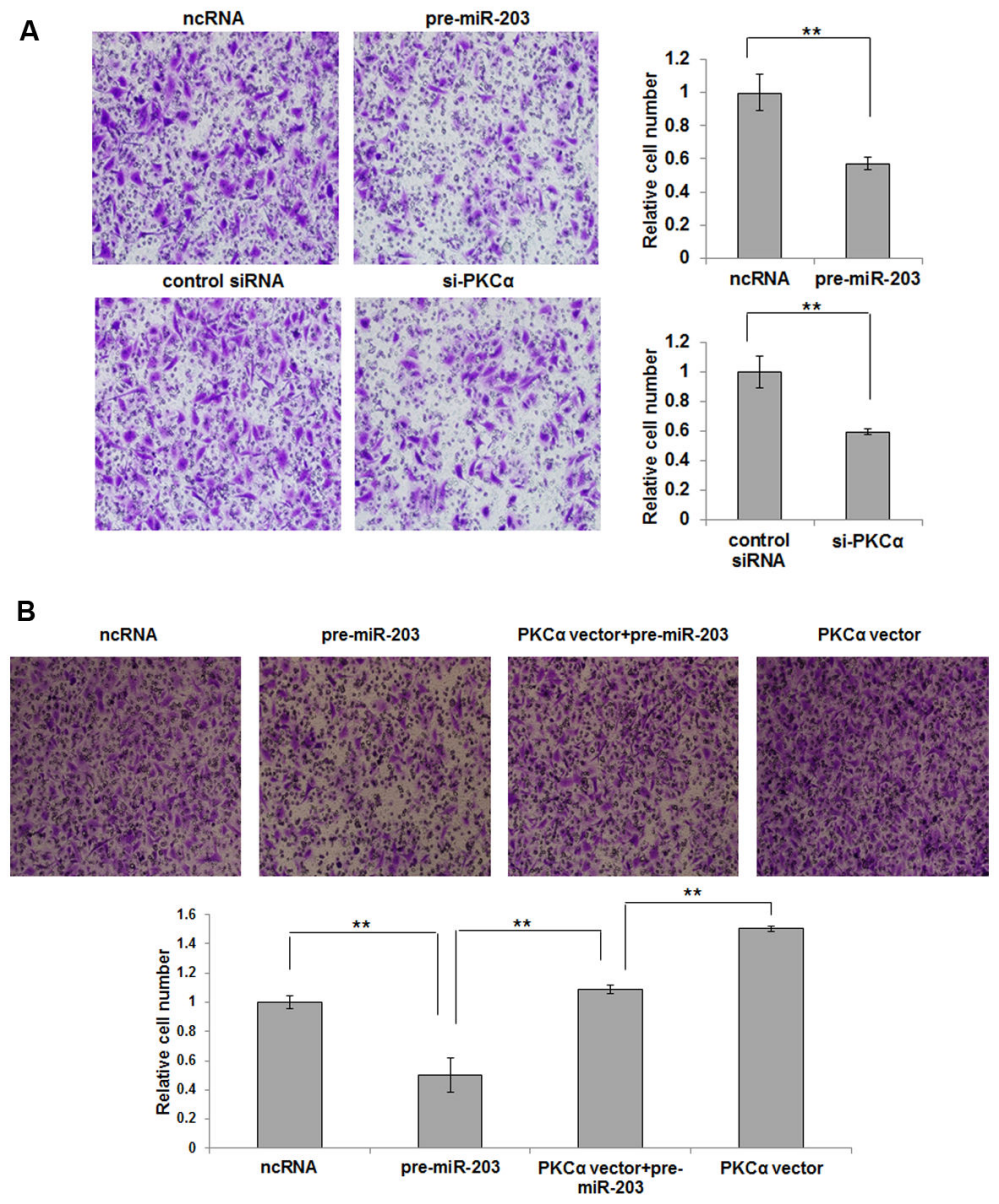
miR-203 inhibits the migration of A549 cells by targeting PKCα. A, Transwell analysis of A549 cells treated with equal doses of scrambled ncRNA or pre-miR-203, or equal doses of control siRNA or si-PKCα. Representative images from three independent experiments are shown in the left panel, and a statistical analysis is shown in the right panel (mean ± S.D.; ** *p*<0.01). B, representative images of Transwell analysis of A549 cells that were transfected with ncRNA, pre-miR-203, pre-miR-203 plus PKCα overexpression plasmid, or PKCα overexpression plasmid alone, are shown in the upper panel, and a statistical analysis is shown in the lower panel (mean ± S.D.; ** *p*<0.01).

We assessed the role of miR-203 in cell migration using the transwell assay. As shown in Figure 6A, the migration rate of A549 cells transfected with pre-miR-203 was significantly decreased when compared to cells transfected with the control ncRNA. Additionally, transfection with si-PKCα remarkably reduced the number of A549 cells that passed through the transwell chamber. Furthermore, when A549 cells were simultaneously transfected with pre-miR-203 and the PKCα overexpression plasmid, PKCα dramatically recovered the migration attenuated by miR-203 (Figure 6B). Taken together, our data suggest that miR-203 probably modulate cell migration by downregulating PKCα.

## Discussion

In addition to lung cancer, PKCα is also a critical factor in many other cancers. It has been found that PKCα, δ, and ι were significantly more abundant in hepatocellular carcinoma (HCC) tissues compared to non-tumor liver tissues [32]. Immunohistochemistry analysis confirmed that above-normal PKCα levels can be found in human HCC [33,34]. When PKCα was introduced into the MCF-7 breast cancer cell line, cell migration and invasion increased [35]. It was reported that treating SK-Hep-1 HCC with antisense PKCα significantly suppressed cell growth, cell migration and invasion [36]. The same results were reproduced using the PKCα/β inhibitor Go6976, which was able to significantly inhibit proliferation, migration, and invasion in poorly differentiated HCC cells. These experiments suggested that PKCα is a practical research direction for understanding cancer development.

miR-203 was reported to act as a tumor-suppressive microRNA, and its expression was downregulated in laryngeal carcinoma cells [37]. Studies from another group showed that miR-203 expression was downregulated in the LNCaP, Du145, PC3, VCaP, and MDA-PCa-2b prostate cancer cell lines [38]. We found that expression of miR-203 in lung cancer tissues was significantly lower than that of the adjacent normal tissues. It has also been shown that miR-203 functions in various cancers. The ectopic expression of miR-203 in prostate cancer cell lines could influence proliferation, apoptosis, and migration [38,39], whereas the overexpression of miR-203 in laryngeal carcinoma cells reduced cell viability and led to a cell cycle arrest in G1 phase [37]. Additionally, expression of miR-203 suppressed cell proliferation and migration in human triple-negative breast cancer cells [40].

Based on computational predictions and experimental validation, we identified PKCα as a novel target for miR-203. The PKCα mRNA levels in lung cancer tissues were found to be higher when compared to the non-tumor tissues; however, miR-203 expression was significantly lower in the tumor tissues. Moreover, we investigated whether certain cellular phenotypes, such as cell proliferation, apoptosis and cell migration, were regulated by the miR-203 mediated regulation of PKCα. We showed that miR-203 negatively regulated cell proliferation and migration by silencing PKCα, and miR-203 could also modulate cell apoptosis. However, siRNA against PKCα only partially phenocopied the apoptotic phenotype elicited by miR-203 overexpression. miR-203 expression may have resulted in more apoptosis than treatments with si-PKCα because multiple apoptosis genes coordinately modulating cell apoptosis may be targeted by miR-203. For example, survivin, a novel anti-apoptosis protein, is regulated by miR-203 [37,38]. Taken together, miR-203 might regulate other genes, although PKCα is certainly an important target of miR-203 due to its effects on other cellular functions.

Because miR-203 functions in cell proliferation and migration by negatively regulating PKCα, the next step would be to search for the downstream target of PKCα; this target may be a common substrate, MARCKS, or the AKT-ERK pathway, which is regulated at least in part by miR-203 [41].

In view of the effects that miR-203 has in modulating cell migration through the inhibition of PKCα, we suggest the generation of an experimental metastasis model to investigate whether the overexpression of miR-203, or the knockdown of PKCα, would suppress metastasis *in vivo*.

In summary, the expression of miR-203 is downregulated in lung cancer cells, and miR-203 can negatively regulate the expression of PKCα. This results in the inhibition of proliferation and the migration of lung cancer cells. Therefore, therapeutic strategies that enhance miR-203 expression or that silence PKCα have the potential to benefit lung cancer patients.
